# Locus Ceruleus Dynamics Are Suppressed during Licking and Enhanced Postlicking Independent of Taste Novelty

**DOI:** 10.1523/ENEURO.0535-23.2024

**Published:** 2024-04-16

**Authors:** Will Fan, Christopher B. Engborg, Natale R. Sciolino

**Affiliations:** ^1^Departments of Physiology and Neurobiology, University of Connecticut, Storrs, Connecticut 06269; ^2^Biomedical Engineering, University of Connecticut, Storrs, Connecticut 06269; ^3^Psychological Sciences, University of Connecticut, Storrs, Connecticut 06269; ^4^Institute for the Brain and Cognitive Sciences, University of Connecticut, Storrs, Connecticut 06269

**Keywords:** fiber photometry, licking, locus ceruleus, norepinephrine, novelty, taste

## Abstract

Attending to salient sensory attributes of food, such as tastes that are new, displeasing, or unexpected, allows the procurement of nutrients without food poisoning. Exposure to new tastes is known to increase norepinephrine (NE) release in taste processing forebrain areas, yet the central source for this release is unknown. Locus ceruleus norepinephrine neurons (LC-NE) emerge as a candidate in signaling salient information about taste, as other salient sensory stimuli (e.g., visual, auditory, somatosensation) are known to activate LC neurons. To determine if LC neurons are sensitive to features of taste novelty, we used fiber photometry to record LC-NE activity in water-restricted mice that voluntarily licked either novel or familiar substances of differential palatability (saccharine, citric acid). We observed that LC-NE activity was suppressed during lick bursts and transiently activated upon the termination of licking and that these dynamics were independent of the familiarity of the substance consumed. We next recorded LC dynamics during brief and unexpected consumption of tastants and found no increase in LC-NE activity, despite their responsiveness to visual and auditory stimuli, revealing selectivity in LC's responses to salient sensory information. Our findings suggest that LC activity during licking is not influenced by taste novelty, implicating a possible role for non-LC noradrenergic nuclei in signaling critical information about taste.

## Significance Statement

Major neuromodulatory systems in the brain respond selectively to various forms of salient information, resulting in a diversity of neuromodulatory states. Novel tastes are a unique form of salience that increase norepinephrine (NE) release in taste-processing brain regions, yet the source for this release is unknown. We recorded calcium activity from the locus ceruleus (LC), the largest central NE nucleus, and found that LC activity was suppressed during licking and enhanced postlicking in a familiarity-independent manner. Furthermore, our findings reveal that unexpected tastants have a minimal impact on LC activity, whereas visual and auditory stimuli elicit robust LC-NE responses. These results provide evidence against LC's involvement in signaling taste novelty and direct attention to other NE subsystems.

## Introduction

Attending to important stimuli in an ever-changing environment permits efficient allocation of neuronal resources. The norepinephrine (NE)-containing neurons in the locus ceruleus (LC) assist the allocation process by engaging key sensory, motor, cognitive, affective, and memory networks upon detection of salient stimuli (Berridge and Waterhouse, 2003; Sara, 2009). Salience, or the potential importance of a stimulus, is determined by multiple factors including novelty, surprise, physical prominence, and association with reward or threat. In monkeys and rodents, unexpected presentation of sensory stimuli (e.g., light, tone, touch) evokes phasic activation in LC neurons, which rapidly diminishes following repeated exposure (Foote et al., 1980; Aston-Jones and Bloom, 1981; Sara and Segal, 1991; Herve-Minvielle and Sara, 1995). In pavlovian and instrumental learning paradigms, the LC is activated by both reward (e.g., water, juice) and punishment (e.g., shock, air puff), as well as by stimuli that predict them (Aston-Jones et al., 1991; Sara and Segal, 1991; Bouret and Sara, 2004; Bouret and Richmond, 2009; Breton-Provencher et al., 2022). Complex sensory stimuli, such as encounters with novel objects during exploration, can also evoke phasic LC responses (Vankov et al., 1995).

The preceding works established the LC as an encoder of general salience, yet it remains unknown whether LC neurons exhibit selectivity toward critical information in different sensory modalities, such as taste. Taste stands apart from other sensory experiences in its intimate connection with ingestive behavior and bodily states. Taste stimuli possess inherent qualities of salience, evoking rapid behavioral and affective reactions (Grill and Norgren, 1978; Steiner, 1979; Grill and Berridge, 1985; Steiner and Glaser, 1995) and providing information about the nutrition and toxicity of the substance ingested (Schier and Spector, 2019; Demi et al., 2021). Novel tastes are particularly salient because their postingestive consequences are unknown. Thus, when animals encounter a novel-tasting food, they tend to sample it with great caution (Barnett, 1958; Carroll et al., 1975; Green and Parker, 1975), a conserved response known as taste neophobia (Rozin, 1976). Depending on the postingestive effects of the novel substance, animals will either familiarize with the taste or develop conditioned taste aversion (Bermúdez-Rattoni, 2004).

The central NE system has been implicated in signaling taste novelty and regulating taste neophobia. For example, the first exposure to a tastant increases NE levels in the insular cortex (IC; Osorio-Gomez et al., 2021) and the basolateral amygdala (BLA; Guzmán-Ramos et al., 2012), which play key roles in novel taste processing (Reilly and Bornovalova, 2005; Lin and Reilly, 2012; Bermudez-Rattoni, 2014; Moraga-Amaro et al., 2014; Lin et al., 2015, 2018). Further, direct infusion of NE in the IC (Rojas et al., 2015) or BLA (Borsini and Rolls, 1984) enhanced taste neophobia, while infusion of the β-adrenergic receptor antagonist propranolol into the IC (Rojas et al., 2015) and BLA (Roozendaal and Cools, 1994) suppressed taste neophobia. The source of this NE release in the IC and BLA is unclear, as these areas receive NE input from both the LC and non-LC nuclei, such as subceruleus, A1, and A2 (Robertson et al., 2013). The LC's sensitivity to general salient information makes it a promising candidate for signaling taste novelty. However, LC activity is also suppressed during food or sweet water consumption (Aston-Jones and Bloom, 1981; Sciolino et al., 2022), raising questions about its responsiveness to novel taste stimuli.

To determine whether LC-NE neurons respond to salient taste stimuli, we used fiber photometry calcium imaging to monitor LC dynamics in water-restricted mice that voluntarily licked novel or familiar tastants with differential palatability [saccharine, citric acid (CA)]. We found that LC-NE activity is suppressed during licking and enhanced postlicking in a manner that is independent of the familiarity of the substance consumed. We also found that long lick bursts were associated with larger increases in LC-NE activity postlicking compared with short lick bursts. Furthermore, our findings reveal that unexpected consumption of tastants has a minimal impact on LC activity, whereas visual and auditory stimuli elicit robust LC-NE responses.

## Materials and Methods

### Animals

All animal procedures were performed in accordance with the [Author University] animal care committee regulations and were in accordance with the *Guide for the Care and Use of Laboratory Animals*. Adult (>P60) male and female mice were used for all experiments. *Dbh^Cre^* mice (Tillage et al., 2020; 033951, Jackson Laboratory; *n *= 12) were maintained on a C57BL/6J background. Mice were housed on a reverse 12 h light/dark cycle with lights off at 9 A.M. (ZT0). All experiments occurred during the dark period of the circadian cycle. Mice had *ad libitum* access to food and water, except during experiments that required water restriction. Mice were group housed until they received surgery.

### Viral preparation

The viruses and titers used are summarized below.

**Table:** 

Plasmid	Virus source	Packaging	Serotype	Titer
pAAV-syn-FLEX-jGCaMP8m-WPRE (Addgene ID 162378)	Janelia Research Campus	Addgene	AAV5	2.4 × 10^13^ GC ml^−1^
pAAV-CAG-FLEX-tdTomato (Addgene ID 28306)	Boyden lab (MIT)	NIEHS Viral Vector Core	AAV5	2.1 × 10^13^ GC ml^−1^
pAAV-syn-FLEX-jGCaMP7f-WPRE (Addgene ID 104488)	Janelia Research Campus	Addgene	AAV9	2.3 × 10^13^ GC ml^−1^

### Stereotaxic surgery

To examine LC responses to gustatory stimuli, *Dbh^Cre^* mice (*n *= 4) received bilateral injections of a 4:1 cocktail of AAVs expressing Cre-dependent GCaMP8m and tdTomato (tdT). To examine LC response to visual/auditory stimuli, a separate cohort of *Dbh^Cre^* mice (*n *= 8) were injected with a 4:1 cocktail of AAVs expressing Cre-dependent GCaMP7f and tdT. Each *Dbh^Cre^* mouse was anesthetized using 4% isoflurane gas and placed in a stereotaxic frame equipped with a digital display (942, Kopf Instruments) and a warming pad (RT-0515, Kent Scientific) to maintain body temperature. Anesthesia was maintained via isoflurane inhalation through a nose cone and was monitored throughout the surgery. An incision was made to expose the skull, and the head was leveled. Two small craniotomies were made above the injection sites using a surgical drill (OmniDrill 35, WPI). A 30-gauge Neuros syringe (7002, Hamilton) loaded with the viral cocktail was slowly lowered to the injection target using the following coordinates relative to the bregma (in mm): −5.45 posterior, ±1.0 lateral, and −3.75 ventral. A microinjection syringe pump (UMP3 UltraMicroPump, WPI) was used to inject 500 nl of the viral cocktail at a rate of 100 nl/min. The needle was left in place for 5 min before being slowly withdrawn, and the incision was sutured close. Mice were returned to their home cage and allowed to recover on a heating pad for at least 24 h.

After at least 3 weeks postinjection, mice underwent a second surgery, in which a custom-made fiber-optic probe (200 μm, 0.39 NA, FT200EMT, Thorlabs) attached to a ceramic ferrule (MM-CON2007-2300-9-BLK, Precision Fiber Products) was unilaterally implanted above the LC using the following stereotaxic coordinates relative to the bregma (in mm): −5.45 posterior, ±0.85 lateral, and −3.71 to −4.31 ventral. Ventral placement of the probe was guided by live fluorescent signals detected through a custom, spectrometer-based photometry system. The excitation light (M470L4, Thorlabs) was launched into a fluorescence cube (DFM1, Thorlabs) containing an excitation filter (ET470/40x, Chroma) and a beam splitter (T495lpxt, Chroma). The excitation light was reflected by a mirror (SM1L20, Thorlabs) into an achromatic fiber port (PAF2-A4A, Thorlabs) and collected by multimode patch cables (M72L02, M83L01, Thorlabs) linked to an implantable optical probe by a mating sleeve (ADAL1-5, Thorlabs). The excitation light from the implantable probe was adjusted to ∼100 µW. Using the same optical fiber and cable, the emitted light was passed through an emission filter (ET500lp, Chroma) before collection by a multimode patch cable. The emitted light was then collected into a spectrometer (Ocean FX, Ocean Insight) and visualized using Ocean View version 2.0.8. Optical probes were secured to the skull using Metabond (S371, S398, and S396, Parkell) and dental acrylic (1406R and 1230CLR, Lang Dental Manufacturing). For pain relief, mice were injected with ketoprofen (5 mg/kg i.p., Covetrus) during surgery, as well as at 24 and 48 h postsurgery. Mice were allowed to recover in their home cage for at least a week before recordings.

### Fiber photometry recordings

In vivo fluorescent signals were recorded from LC^GCaMP/tdT^ mice using a spectrally resolved, fiber photometry system (Meng et al., 2018; Mazzone et al., 2020; Sciolino et al., 2022). A beam of excitation laser (488 nm, OBIS 488LS-20, Coherent) was launched into a fluorescence cube (DFM1, Thorlabs), reflected by a dichroic mirror (ZT488/561rpc-UF1, Chroma), and focused by an achromatic fiber port (PAFA-X-4-A, Thorlabs) onto the core of a multimode patch cable (M83L01, Thorlabs). The distal end of the patch cable was connected by a quick release interconnect (ADAL3, Thorlabs) to an optical probe made with a multimode fiber (200 µm, 0.39 NA, FT200EMT, Thorlabs) and a ceramic ferrule OD (MM-CON2007-2300, Precision Fiber Products). The emitted fluorescence was collected by the same optical probe and patch cable, passed through the same dichroic mirror, and filtered through an emission filter (ZET 488/561m) before being collected by a fiber port (PAF2S-11A, Thorlabs) and launched into a spectrometer (Ocean FX, Ocean Optics) through a multimode patch cable (M200L02-A, Thorlabs). Time-lapsed fluorescence emission spectra were visualized using Ocean View version 2.0.8 at an acquisition rate of 25 frames/s, with an integration time of 31 ms. The spectrometer was triggered by a 25 Hz transistor–transistor logic (TTL) signal that was generated by the output module (DIG-26TTL, Med Associates), which allows alignment of the photometry data with behavioral events.

### Fiber photometry data analysis

The raw photometry signals were preprocessed using a custom-written R script to linearly unmix the partially overlapping GCaMP and tdT signal, as previously described (Meng et al., 2018). To remove movement artifacts from the GCaMP traces (e.g., photon loss caused by tissue movement or bending of the patch cable during mouse movement), we used the ratio between the unmixed GCaMP and tdT signals (fluorescent ratio) for all subsequent analyses. The fluorescent ratio traces were analyzed using custom-written scripts in MATLAB (version R2022a, MathWorks). All traces were detrended to correct for signal decay due to photobleaching. %dF/*F* values were calculated by normalizing fluorescent ratios to the means of the baseline periods. For specifications of the baseline periods, see below, Behavioral paradigms for fiber photometry.

### Behavioral paradigms for fiber photometry

All behavioral experiments were conducted in operant chambers (ENV-370W, Med Associates) that were placed in a sound-attenuating cubicle (ENV-017M, Med Associates). The cubicle had a ventilation fan, which generated 62 dB background noise and was illuminated with a dim, red LED (5 lux). Behavior was recorded though a monochrome camera (BFS-U3-28s5M-C, Wilco Imaging), which was triggered by a 25 Hz TTL signal that was generated by the Med Associates output module. Custom-written MedPC programs were used to control the camera, lickometer, and light and sound stimuli and acquire behavioral timestamps.

#### Licking novel and familiar substances

Across several days prior to the recording, LC^GCaMP/tdT^ mice were habituated to 23 h of water deprivation and trained to consume water from a sipper bottle wired with a contact lickometer (ENV-250, Med Associates). A lick event was registered when a mouse contacts both the grid floor and the spout. On the first day of habituation, water-deprived mice were given access to the sipper until at least 300 licks were detected. Mice were then returned to the home cage and given 1 h of *ad libitum* access to water. On the subsequent days of habituation (3–5 d), mice were given 15 min access to the sipper each day, followed by 1 h of home cage water. This training continued until a stable drinking behavior (>800 licks/session) was established. The recordings for each experiment occurred across 3 d, in the following order: water baseline, novel tastant (first exposure), and familiar tastant (second exposure). Each recording began with 5 min of baseline, during which access to the sipper was blocked by a barrier. The barrier was removed at the end of the baseline, and mice had access to the sipper for 15 min during the experiment. Mice were returned to the home cage and given 1 h of *ad libitum* water access. Mice were kept on water restriction between the two sets of experiments, involving 0.2% sodium saccharine or 10 mM CA, ran in that order. For analysis, the processed photometry signals were normalized to the 5 min presession baseline. A lick burst was defined as a sequence of at least three licks, with an interlick interval of <1 s. Traces were aligned to the onset and offset of the lick bursts. The mean dF/*F* was calculated during and following (0–1 s after burst offset) the lick bursts. To examine the magnitude of LC dynamics as a function of lick burst order and length, we focused our analysis on saccharine sessions and omitted the CA sessions, which had low total lick burst number.

#### Unexpected, brief taste exposures

LC^GCaMP/tdT^ were trained to consume water for 5 consecutive days from a valve-controlled gravity perfusion system (VC3-4PG, ALA Scientific Instruments), which had four fluid reservoirs that each was connected via 1/16″ PVC tubing to a branch of polyimide manifold (MMF-4, ALA Scientific Instruments). The four fluid outlets were in separate, parallel paths and inserted flush into a custom-made metal spout (ID = 3.0 mm) connected to the lickometer. Fluid flow from each reservoir was regulated by individual fast-opening (<20 ms) solenoid pinch valves, which were opened by TTL signal from the Med Associates output module, allowing licking behavior to be coupled to fluid delivery. During the training session (20–30 min), one of the pinch valves opened for 50 ms at every fifth lick to release 3 µl of water (rinse). On the recording day, the fluid delivery program was modified, such that after every 10 water rinses, mice received five licks of a tastant, including 0.2% sodium saccharine or 10 mM CA, presented in a shuffled order to minimize the mouse's expectations regarding which tastant would be delivered. Each of the five licks of tastant was interleaved by a dry lick, so that the tastant presentation would be brief, lasting ∼1 s if licking was uninterrupted. The recording session was 20 min, beginning with a 5 min baseline where the spout was inaccessible, followed by 15 min of sipper access. For analysis, the processed photometry signals were normalized to the 5 min presession baseline. Traces were aligned to the onset of each tastant presentation. The peak dF/*F* was calculated before (2–0 s relative to the onset) and during (0–1 s relative to the onset) the tastant presentations.

#### Visual and auditory exposures

LC^GCaMP/tdT^ mice were habituated for 3 d to being tethered in the operant chamber. On the recording day, mice were presented with 10 light and sound stimuli in shuffled order. To minimize the mouse's expectations regarding which sensory stimulus would be delivered, we used a varying interstimulus interval (90–120 s). The light stimulus was briefly presented (50 ms) and generated by a house light (55 lux) in the operant box. The sound stimulus was also briefly presented (250 ms, 4 kHz, 100 dB) and generated by a speaker (ENV-224CM, Med Associates), which was mounted on the side of the cubicle. The processed photometry signals (see above, Fiber photometry data analysis) were aligned to the onset of each stimulus. The LC-NE response to each stimulus was normalized to a 10 s baseline period (located −15 to −10 s relative to stimulus onset). The peak dF/*F* was calculated before (2–0 s relative to stimulus onset), during, and after (2–4 s relative to stimulus onset) the presentation of each sensory stimulus.

### Tissue collection

Adult mice were deeply anesthetized using a cocktail of ketamine (500 mg/kg i.p., 71069, Covetrus) and xylazine (50 mg/kg i.p., 61035, Covetrus). Anesthetized mice were perfused transcardially with a phosphate-buffered saline (PBS), followed by 4% paraformaldehyde in PBS (PFA/PBS). The brains were then postfixed overnight through immersion in 4% PFA/PBS at 4°C on a shaker. The tissue was then cryoprotected in 30% sucrose in PBS followed by embedding in a cryostat embedding medium (O.C.T., Thermo Fisher Scientific). Brains were cryosectioned into 40 µm coronal sections. Slices were transferred to a cryoprotectant tissue storage solution and stored at −80°C.

### Immunohistochemistry

Brain sections were rinsed in PBS and blocked in 5% normal goat serum (made in 0.1% Triton PBS) for 1 h at room temperature on a rotary shaker. Tissues were incubated overnight at 4°C in primary antibodies, including chicken anti-GFP, rabbit anti-DsRed, and mouse anti-TH. The next day, tissues were washed in 0.1% Triton X-100 and incubated at room temperature for 2 h in secondary antibodies, including goat anti-chicken Alexa 488, goat anti-rabbit Alexa 568, and goat anti-mouse Alexa 633. The brain sections were mounted onto slides and a coverslip with a Prolong Diamond Anti-Fade mounting medium with 4′,6-diamidino-2-phenylindole (P36971, Thermo Fisher Scientific) was applied. The antibodies used are summarized below.

**Table:** 

Antibody	Species	Dilution	Source	Catalog #	LOT #
EGFP	Chicken	1:10,000	Abcam	AB13970	GR3361051-14
dsRed	Rabbit	1:1,000	Takara	632496	2103116, 2210019
TH	Mouse	1:500	GeneTex	GTX10372	822104279
Alexa Fluor 488 anti-chicken	Goat	1:1,000	Thermo Fisher Scientific	A11039	2304258
Alexa Fluor 568 anti-rabbit	Goat	1:1,000	Thermo Fisher Scientific	A11036	2273773
Alexa Fluor 633 anti-mouse	Goat	1:1,000	Thermo Fisher Scientific	A21052	2304276

### Digital image processing

Images of immunofluorescently labeled sections were collected using a 40× objective on a confocal microscope (A1R, Nikon). Fiji version 1.53c (Schindelin et al., 2012) was used to convert the z-stacks to a maximum intensity projection image. Images were modified only by adjusting the brightness and contrast across the entire image to optimize the fluorescence signal. Anatomical location was confirmed by reference to a mouse brain atlas (Paxinos and Franklin, 2019).

### Statistical analyses

Repeated measures ANOVAs and paired-samples *t* tests were used to determine differences between groups. Bonferroni’s post hoc tests were used as appropriate. Significance was set at *p *< 0.05 for all analyses. Linear regression was used to quantify burst order. All predictions were two-tailed and expressed as mean ± standard error (SEM). Statistics and sample size are listed in the figure legends. All analyses were performed using Prism version 9 (GraphPad Software).

## Results

### LC-NE activity is suppressed during licking and enhanced postlicking independent of tastant familiarity

**Figure 1.**
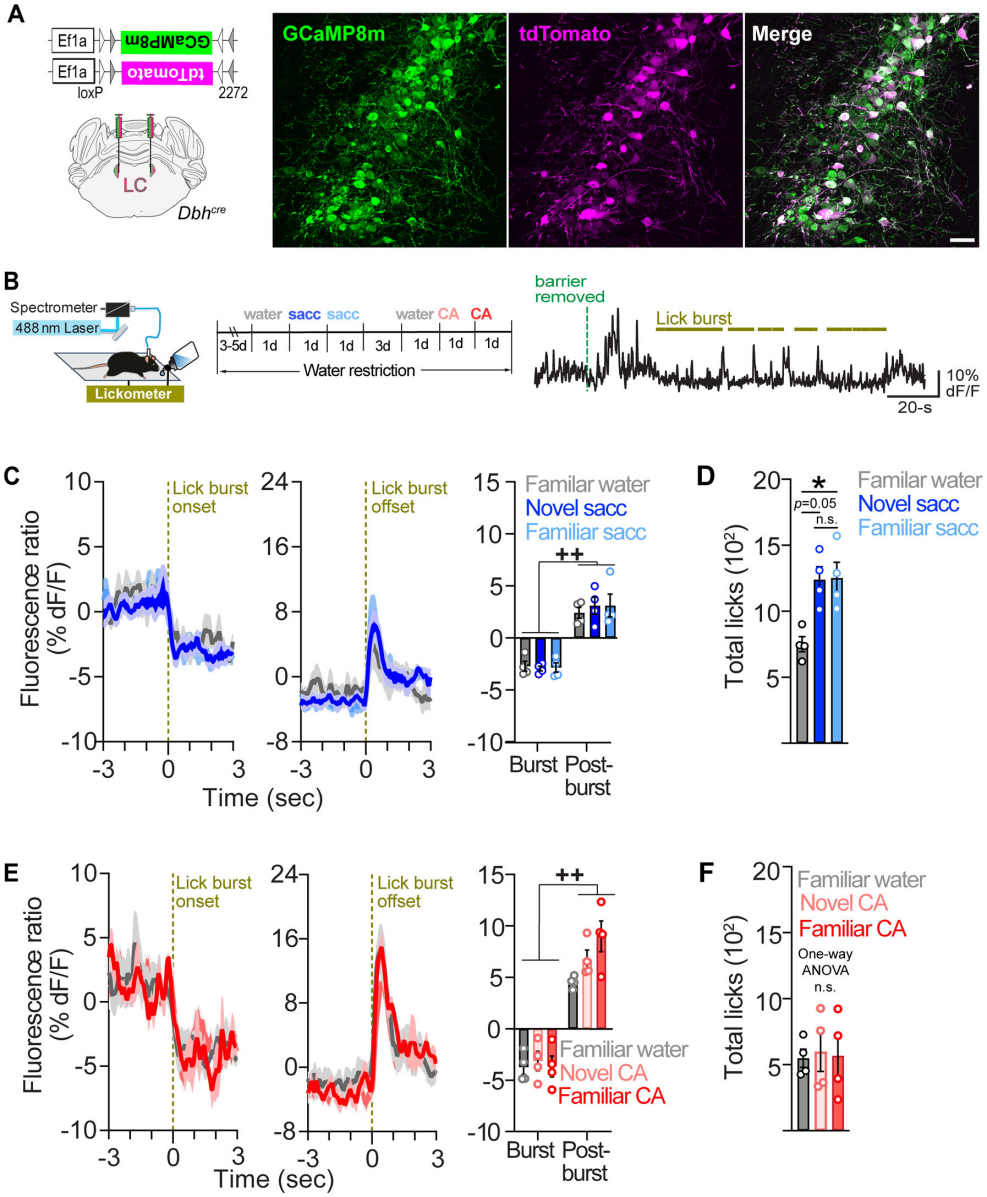
LC-NE activity is suppressed during licking and transiently activated postlicking regardless of the substance consumed or its familiarity. ***A***, Left: Cre-dependent viral genetic approach for coexpression of GCaMP8m and tdT in LC-NE neurons. Right: Coronal section of the LC from an LC^GCaMP/tdT^ mouse immunostained for GCaMP8m (GFP) and tdT (dsRed). Scale bar, 50 µm. ***B***, Left. Schematic of in vivo fiber photometry setup and lickometer. Middle: Timeline of the photometry experiments during saccharine (sacc.) and CA exposures. Right: Fluorescence ratio from a representative water-restricted LC^GCaMP/TdT^ mouse during its first encounter with saccharine. Lick bursts are shown above the trace. ***C***, Average fluorescence ratio aligned to the onset (left) and offset (middle) of lick bursts during the novel and familiar saccharine and water exposure. Right: Quantification of the mean fluorescence ratio during licking-related events. Two-way repeated measures ANOVA: Epoch main effect (*F*_1.00, 3.00 _= 43.52; *p *= 0.01). Epoch effect, ^++^*p *< 0.01. Familiarity main effect (*F*_1.30, 3.90 _= 0.18; *p *= 0.76). Epoch* *×* *familiarity interaction (*F*_1.67, 5.00 _= 0.53; *p *= 0.59). Data are mean ± SEM. *n *= 4 LC^GCaMP/tdT^ mice. ***D***, Average total licks across the 15 min session during the novel and familiar exposure to saccharine. One-way repeated measures ANOVA (*F*_1.27, 3.80 _= 14.21; *p *= 0.02). Bonferroni’s post hoc test, *p < 0.05. n.s., not significant. Data are mean ± SEM. *n *= 4 LC^GCaMP/tdT^ mice. ***E***, Average fluorescence ratio aligned to the onset (left) and offset (middle) of lick bursts during the novel and familiar exposure to 10 mM CA in water-restricted LC^GCaMP/TdT^ mice. Right: Quantification of the mean fluorescence ratio during licking-related events. Two-way repeated measures ANOVA: Epoch main effect (*F*_1.00, 3.00 _= 50.76; *p *= 0.01). Epoch effect, ^++^*p *< 0.01. Familiarity main effect (*F*_1.38, 4.15 _= 8.91; *p *= 0.04). Epoch_ _×_ _familiarity interaction (*F*_1.33, 4.00 _= 3.67; *p *= 0.13). Data are mean ± SEM. *n *= 4 LC^GCaMP/tdT^ mice. ***F***, Average total licks across the 15 min session during the novel and familiar exposure to CA. One-way repeated measures ANOVA: *F*_1.13, 3.40 _= 0.09; *p *= 0.81. Data are mean ± SEM. *n *= 4 LC^GCaMP/tdT^ mice. Extended Data Figure 1-1 shows optical fiber placement.

To examine LC's role in signaling taste novelty, we first tested if LC-NE neurons exhibit increased activity during the consumption of a novel taste compared with a familiar one. We used fiber photometry (Meng et al., 2018; Sciolino et al., 2022) to monitor GCaMP8m (Y. Zhang et al., 2023) tdT fluorescence activity in LC-NE neurons of mice expressing Cre recombinase under the noradrenergic-specific promoter dopamine-beta hydroxylase (*Dbh^cre^*, hereafter termed LC^GCaMP/tdT^ mice; Fig. 1*A*; Extended Data Fig. 1-1). Water-restricted LC^GCaMP/tdT^ mice were first habituated to drinking from the lickometer, and then fiber photometry recordings were performed while mice were licking water (familiar control) or a preferred tastant (0.2% saccharine; Bachmanov et al., 2001) that was either novel (first exposure) or familiar (second exposure; Fig. 1*B*). We observed a suppression of LC-NE neurons during lick bursts (periods of uninterrupted licking) and a transient activation immediately following the cessation of lick bursts (Fig. 1*B*). Sensitivity to taste novelty would be indicated if LC-NE neurons are more active in response to novel tastants compared with familiar tastant and water. However, we observed no difference in the magnitude of LC suppression and postburst activation when mice consumed novel saccharine, familiar saccharine, and water (familiarity_ _×_ _epoch interaction: *F*_1.67, 5.00 _= 0.53, *p *= 0.59; familiarity main effect: *F*_1.30, 3.90 _= 0.18, *p *= 0.76; Fig. 1*C*), which fails to support the hypothesis that LC activity signals taste novelty. Assessment of the licking behavior revealed that LC^GCaMP/tdT^ mice drank more saccharine, regardless of its novelty, compared with the water control, confirming saccharine as a preferred tastant (Fig. 1*D*). To determine if the familiarity of a less palatable tastant would influence consummatory-related LC dynamics, we next measured the activity of LC-NE neurons as mice consumed CA (10 mM). LC-NE neurons were also suppressed during lick bursts and activated following lick bursts for CA (Fig. 1*E*). We observed a significant main effect for familiarity (*F*_1.38, 4.15 _= 8.91; *p *= 0.04; Fig. 1*E*), although post hoc tests were not significant and revealed a trend for a larger postlicking response to familiar CA compared with water (*p *= 0.10). A subsequent analysis of the behavior revealed that LC^GCaMP/tdT^ mice drank a comparable amount of CA compared with water during both exposures (Fig. 1*F*), suggesting a similar palatability between CA and water. Taken together, our results demonstrate that LC-NE neurons are inhibited during licking and activated briefly upon licking termination regardless of the familiarity of the substance consumed.

10.1523/ENEURO.0535-23.2024.f1-1 Extended data Figure 1-1 **Fiber photometry recording sites relative to the LC.** The location of the implanted optical fiber in the hindbrain of LC^GCaMP/TdT^ mice. The ventral tip of the optical fiber is indicated by a dot for each subject. Mice from the taste experiment are shown as green dots (*n* = 4). Mice from the visual/auditory experiment are shown as blue dots (*n* = 6), which does not include two subjects due to technical issues with tissue collection that prevented the precise localization of the optical probe. The schematic shows coronal sections adapted from a mouse reference atlas (Paxinos and Franklin, 2019) using coordinates relative to bregma (mm). Mice from the different experiments are shown on the opposite hemisphere of the schematic for illustrative purposes. Download Extended data Figure 1-1, TIF file.

### The magnitude of LC rebound after licking is positively correlated with the length of lick bursts, but not with LC suppression during licking

**Figure 2.**
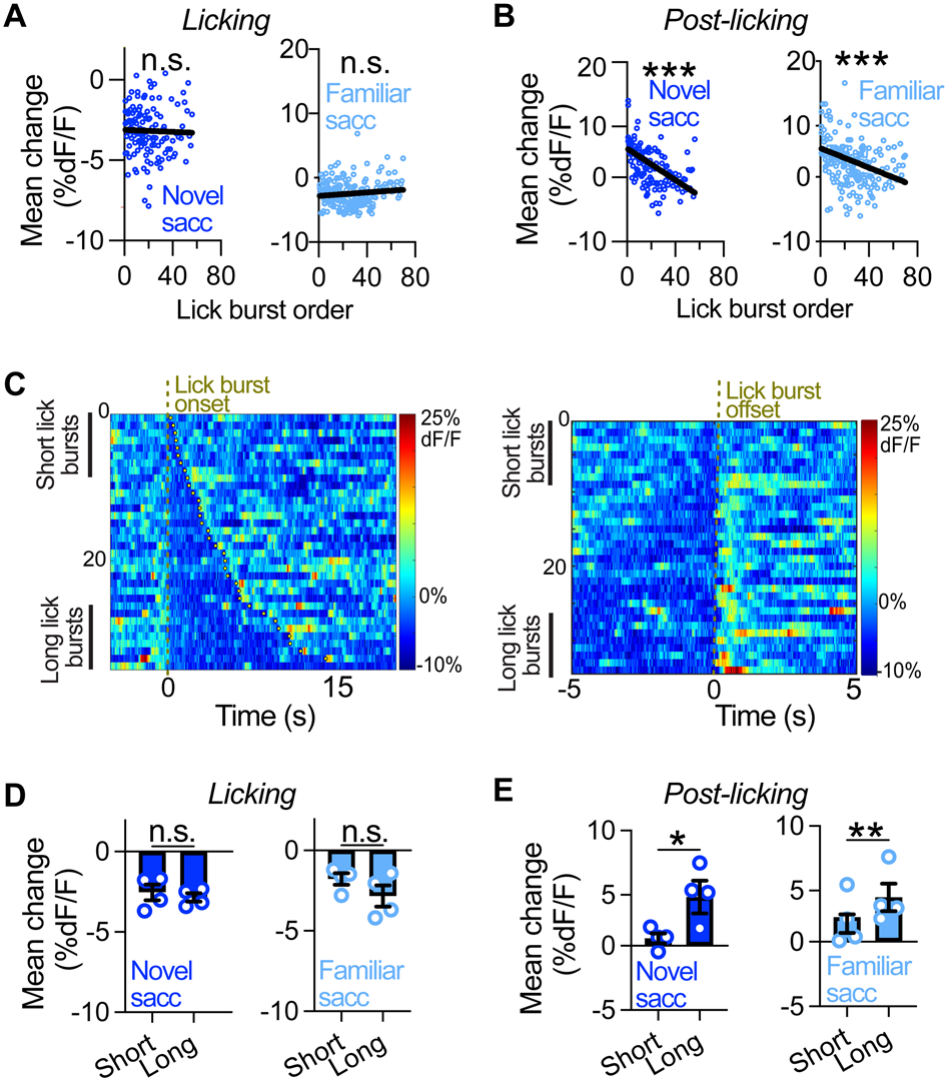
Inhibition of LC-NE neurons during consumption is independent of the lick burst length, whereas LC activation postconsumption is larger in magnitude for long versus short lick bursts. ***A***, Linear regression of the licking-induced change in the fluorescence ratio across the successive lick bursts during the novel (left) and familiar exposure (right) to saccharine in water-deprived LC^GCaMP/tdT^ mice. Saccharine licking response during the novel exposure (slope = −0.0031 ± 0.0092; *R*^2 ^= 0.0008; *F*_1,132 _= 0.11; *p *= 0.74) and familiar exposure (slope = 0.0137 ± 0.0084; *R*^2 ^= 0.0136; *F*_1,191 _= 2.63; *p *= 0.11). ***B***, Linear regression of the postlicking change in the fluorescence ratio across successive lick bursts during the novel (left) and familiar exposure (right) to saccharine. Saccharine postlicking response during the novel (slope = −0.1227 ± 0.0192; *R*^2 ^= 0.2365; *F*_1,132 _= 40.88; ***p < 0.0001) and familiar exposure (slope = −0.0754 ± 0.0162; *R*^2 ^= 0.1018; *F*_1,191 _= 21.66; ***p < 0.0001). ***C***, Heatmap from a representative water-restricted LC^GCaMP/TdT^ mouse showing the fluorescence ratio aligned to licking onset (left) and offset (right), where lick bursts are sorted by their length during the novel saccharine exposure. ***D***, Average licking-induced change in the fluorescence ratio for long versus short lick bursts during the novel (left) and familiar (right) saccharine exposure. Paired sample *t* test: licking during novel (*t*_3 _= 1.35; *p *= 0.27) and familiar saccharine exposure (*t*_3 _= 1.84; *p *= 0.16). Data are mean ± SEM. *n *= 4 LC^GCaMP/tdT^ mice. ***E***, Average postlicking change in the fluorescence ratio for long versus short lick bursts during the novel (left) and familiar (right) saccharine exposure. Paired sample *t* test: postlicking during the novel (*t*_3 _= 4.84; *p = 0.02) and familiar (*t*_3 _= 7.36; **p < 0.01) saccharine exposure. Data are mean ± SEM. *n *= 4 LC^GCaMP/tdT^ mice.

Alterations in the perceived familiarity of tastants can occur within a single drinking session (Monk et al., 2014). To determine if licking-related LC dynamics change throughout the session, we performed a linear regression to compare LC responses across successive lick bursts during both novel and familiar exposures to 0.2% saccharine in water-restricted LC^GCaMP/tdT^ mice. We found that the magnitude of LC-NE suppression during licking was similar across successive lick bursts for both saccharine exposures (Fig. 2*A*). In contrast, the magnitude of LC-NE activation postlicking was significantly attenuated across successive lick bursts for both saccharine exposures (Fig. 2*B*). Given that longer lick bursts are known to occur early in the session when mice are thirstier (Naneix et al., 2020), we next sought to determine if the magnitude of LC-NE dynamics during licking-related events would change as a function of lick burst length. To do this, we sorted lick bursts by their length and compared LC responses for long (first quartile) versus short (fourth quartile) lick bursts (Fig. 2*C*). The licking-related suppression of LC-NE activity was similar for long and short lick bursts during both saccharine exposures (Fig. 2*D*). In contrast, the postlicking-related increase in LC-NE activity was larger during long lick bursts compared with short bursts during both saccharine exposures (Fig. 2*E*). Collectively, our findings demonstrate that the postlicking increase in LC-NE activity varies as a function of lick burst length and order, but the licking-related LC suppression is not affected by these measures.

### LC-NE neurons are activated by brief exposure to salient visual and auditory stimuli, but not taste stimuli

**Figure 3.**
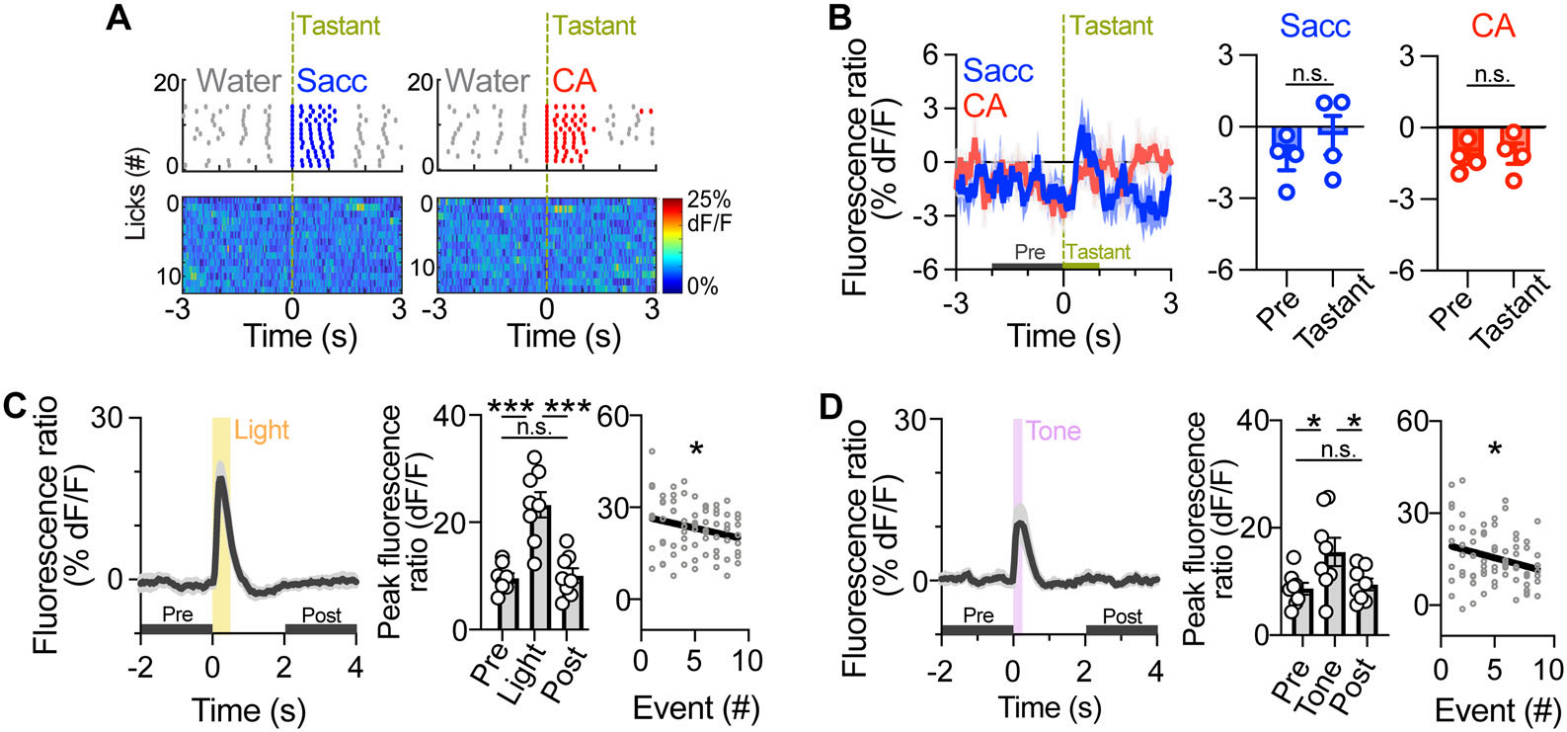
Unexpected consumption of tastants does not significantly alter LC activity, whereas unexpected visual and auditory stimuli elicit LC-NE activation. ***A***, Top: Raster plot of fluid-paired licks aligned to the onset of brief, unexpected presentations of 0.2% saccharine (sacc.; left) and 10 mM CA (CA; right) from a water-restricted LC^GCaMP/TdT^ mouse. Licks of saccharine and CA are colored blue and red, respectively. Water rinses are colored gray. Dry licks are not shown. Bottom: Heatmap shows the fluorescence ratio aligned to the onset of licking unexpected saccharine (left) and CA (right) across consecutive trials in the session. ***B***, Left: Average fluorescence ratio aligned to the onset of unexpected tastants in water-restricted LC^GCaMP/TdT^ mice. Right: Average fluorescence ratio before and during lick bursts of unexpected tastants. Paired sample *t* test: Saccharine (*t*_3 _= 1.88; *p *= 0.16) and CA (*t*_3 _= 0.77, *p *= 0.50). n.s., not significant. Data are mean ± SEM. *n *= 4 LC^GCaMP/tdT^ mice. ***C,D***, Left: Average fluorescence ratio aligned to the onset of an unexpected flash of light (***C***) and pulse of sound (***D***) in LC^GCaMP/TdT^ mice. Middle: Average peak fluorescence ratio during visual (***C***)- or auditory (***D***)-related events. One-way repeated measures ANOVA for light (*F*_1.27, 8.92 _= 51.26; *p *< 0.0001) and sound (*F*_1.14, 7.99 _= 12.21; *p *= 0.01). Bonferroni’s post hoc test, ***p < 0.001, *p < 0.05. Data are mean ± SEM. *n *= 8 LC^GCaMP/tdT^ mice. Right: Peak fluorescence ratio across consecutive exposures to light (***C***) and sound (***D***) in LC^GCaMP/tdT^ mice. Linear regression for light (slope = −0.7812 ± 0.3788; *R*^2 ^= 0.05727; *F*_1,70 _= 4.25; *p = 0.04) and sound (slope = −0.9339 ± 0.4164; *R*^2 ^= 0.06703; *F*_1,70 _= 5.03; *p = 0.03). Extended Data Figure 1-1 shows the optical fiber placement.

Salient sensory information, including visual, auditory, and somatosensory stimuli, robustly activate LC neurons (Foote et al., 1980; Aston-Jones and Bloom, 1981), but this response was not observed during tasting (Aston-Jones and Bloom, 1981; Sciolino et al., 2022). One possibility for the absence of LC activation by taste is that tastants accessed repeatedly and voluntarily are predictable, unlike passively delivered stimuli of other sensory modalities. To determine if brief and unpredicted taste exposures would activate LC neurons, we used a programmed taste delivery paradigm (Stapleton et al., 2006; Roussin et al., 2012) to deliver tastants to water-restricted LC^GCaMP/tdT^ mice (Extended Data Fig. 1-1). Mice were first trained to “dry” lick a spout five times to receive a drop of water (3 µl rinse). Following training, photometry recordings were collected while mice received five licks of a tastant, either saccharine (0.2%) or CA (10 mM), after every 10 rinses, with the tastant identity shuffled. The onset of tastant presentation varies temporally due to the variable gap periods between lick bursts. We found that the suppression of LC-NE activity during consumption of the unexpected tastants was not changed compared with the water rinse period (Fig. 3*A,B*), suggesting that unexpected taste exposures have no significant effect on LC-NE dynamics. To confirm the known finding that other sensory stimuli (e.g., light, sound) activate LC neurons (Foote et al., 1980; Aston-Jones and Bloom, 1981; Sara and Segal, 1991; Herve-Minvielle and Sara, 1995), we presented LC^GCaMP/tdT^ mice with flashes of light (0.5 s) or pulses of sound (0.25 s) in shuffled order. As expected, we observed a rapid, transient activation of LC-NE neurons to the visual and auditory stimuli, and the magnitude of these responses was significantly attenuated across repeated trials (Fig. 3*C,D*), consistent with previous findings (Foote et al., 1980; Aston-Jones and Bloom, 1981; Sciolino et al., 2022). Collectively, our results demonstrate that LC-NE neurons are not activated by unexpected consumption of taste stimuli.

## Discussion

Novel taste, a unique form of salient information, engages the cholinergic, dopaminergic, and noradrenergic systems to facilitate novelty detection, cautious sampling, and memory formation (Osorio-Gómez et al., 2018; Gil-Lievana et al., 2022). The NE system has been shown to regulate taste neophobia by modulating the IC and BLA (Borsini and Rolls, 1984; Roozendaal and Cools, 1994; Guzmán-Ramos et al., 2012; Rojas et al., 2015; Osorio-Gomez et al., 2021), yet the specific NE subsystem(s) responsible for this action remains unclear. The LC-NE system presents as promising candidate for signaling taste novelty, as the LC innervates both the IC and BLA (Robertson et al., 2013). In the present study, we found that LC-NE dynamics, characterized by a suppression during lick bursts and rebound activation following lick bursts, is not modulated by taste novelty. A larger postlicking rebound was observed with longer lick durations, while the response magnitude during lick bursts stayed the same. Finally, unexpected consumption of tastants did not activate LC-NE neurons, contrasting with their established response to visual and auditory stimuli. These findings suggest that the LC-NE system plays a limited role in signaling taste novelty and redirects attention to non-LC sources of NE.

The LC's insensitivity to novelty and unpredictability of taste implies fundamental distinctions between taste and other modalities (sight, sound, touch, and smell). Conceptually, taste has salient properties relevant to nutritional quality or food toxicity, whereas other stimuli that evoke salient responses are generally associated with the presence of reward or threat in the environment (Breslin, 2013). This suggests a potential rivalry between taste and other sensory modalities, as focusing on food and internal state requires diverting attention from the environment and vice versa. Thus, being alerted by a taste represents a distinct behavioral state compared with being alerted by an odor or sound. Hence, it is plausible that taste and other salient external stimuli would differentially modulate LC-NE dynamics.

The magnitude of LC responses may be dependent on the physical intensity of the sensory stimulus (Grant et al., 1988). Therefore, a more concentrated taste stimulus could potentially elicit an excitatory response in the LC, overcoming the suppression observed during licking. The sensory stimuli employed in this study are of moderate intensity; specifically, the taste stimuli are nonaversive (e.g., voluntarily consumed), and the visual and auditory stimuli do not elicit startle or freezing responses. Hence, LC's transient excitatory responses to the light and tone likely reflect attention shifts rather than stress responses (Abercrombie and Jacobs, 1987). The absence of taste responses in LC may also be attributed to the self-administered nature of taste stimuli. Even in our programmed tastant delivery paradigm, where mice were unaware of the timing and type of tastant delivered, they could anticipate the reception of taste when engaged in licking. To deliver taste stimuli in a completely unexpected manner would involve passively infusing tastants into the oral cavity (Grill and Norgren, 1978). This approach enables testing substances that animals naturally reject, such as innately unpalatable or aversively conditioned tastants, which may potentially influence LC activity.

The suppression of LC activity during consumption is thought to reflect a temporary diminishment in arousal (Sciolino et al., 2022), which may be necessary for stabilizing endogenous rhythmic behavior such as licking (Aston-Jones and Bloom, 1981). While these dynamics have been observed, the nature and source of the inhibitory input remain elusive. In our study, the initiation and termination of LC suppression align closely with those of a lick burst, indicating a link between the inhibitory drive and licking activity. The inhibitory signal is likely constant during ingestion, as the magnitude of LC suppression during short and long lick bursts was similar. LC's suppression during feeding, in an earlier study, was characterized as a “gustatory response” with no relation to the licking activity (Aston-Jones and Bloom, 1981), as the observed inhibition did not appear to fluctuate with individual licks (Aston-Jones and Bloom, 1981). However, it is conceivable that the inhibitory input to the LC during licking arises from premotor region(s) that drive licking rhythms. This premotor signal may arise from brain structure(s) such as the lateral hypothalamus and/or the central amygdala, which send GABAergic projections to the LC (Marino et al., 2020; Liu et al., 2021) and are known to modulate motor circuits for licking/feeding (Travers et al., 1997; Mascaro et al., 2009; Zheng et al., 2022).

The dynamics of the LC upon consumption termination have not been explored before. We found that LC-NE neurons recover abruptly from suppression, followed by a phasic activation. In a recent study involving the consumption of solid food, LC neurons exhibited rapid suppression at the onset of feeding, followed by a gradual return to baseline as feeding continued (Sciolino et al., 2022). This apparent slow recovery may arise from averaging LC activity across feeding bouts with variable durations. The observed increase in LC calcium activity postlicking could be driven by an excitatory input or intrinsic properties of LC-NE neurons. The former is more likely as slice electrophysiology studies in rats have provided limited evidence for the existence of postinhibitory rebound in the LC (Williams et al., 1984; X. Zhang et al., 2010). Further, we observed a correlation between the magnitude of LC-NE activation and lick burst duration, but the significance of this finding is unknown. The variation in the magnitude of postlicking LC responses observed in the present study is likely accounted for by differences in licking pattern between substances and familiarity. A key focus in LC research revolves around the functional distinction between tonic and phasic modes of activation (Rajkowski et al., 1994; Aston-Jones and Cohen, 2005; Devilbiss and Waterhouse, 2011; Vazey et al., 2018; Grella et al., 2019). In particular, phasic activation has been associated with focused task performance (Aston-Jones and Cohen, 2005) and stimulus-driven cognitive shifts (Bouret and Sara, 2005), exerting profound influences on the brain network function (Rajkowski et al., 1994; Devilbiss and Waterhouse, 2011; Neves et al., 2018; Vazey et al., 2018; Grella et al., 2019; Durán et al., 2021). We speculate that LC phasic activation at the end of each brief feeding bout facilitates a transient attention shift—from the food to the environment—thereby maintaining a state of vigilance during the consummatory process. This is consistent with our observation that when a lick burst terminates, mice retract their head from the sipper port and reorient their head away from the sipper. Understanding the causal role of LC's activity during the consummatory action sequence on cortical dynamics and arousal responses will be an exciting direction for future investigation.

LC's insensitivity to taste novelty, together with findings implicating a role for NE in novel taste processing (Borsini and Rolls, 1984; Roozendaal and Cools, 1994; Guzmán-Ramos et al., 2012; Rojas et al., 2015; Osorio-Gomez et al., 2021), suggests that other central NE subpopulations may be involved in signaling taste novelty. Taste-processing regions such as the IC and BLA receive dense innervation from both the LC and a subpopulation of non-LC NE neurons defined by the transient expression of the transcription factor *Hoxb1* (Robertson et al., 2013). *Hoxb1*-derived NE neurons are dispersed in the adult ventral subceruleus, A5, A2, and A1 nuclei. Central autonomic brain regions such as the hypothalamic nuclei, central amygdala, bed nucleus of the stria terminalis, and nucleus of the solitary tract (Saper, 2002) receive dense innervation from *Hoxb1*-derived NE neurons (Robertson et al., 2013). Given the tight interconnection between taste and central autonomic pathways (Saper, 2002), *Hoxb1*-derived NE neurons are well positioned to signal critical properties about taste and complement input from the LC (Robertson et al., 2013; Chen et al., 2019). Identifying the responsive central NE subpopulation(s) to taste stimuli in future studies will offer insights into the functional divisions of the central NE system.

The lack of rapid modulation by taste novelty does not preclude the LC from participating in novel taste processing in various ways. For example, subtle changes in LC activity during exposure to novel tastants may be difficult to discern from our small sample size. Of note, prior studies using microdialysis found an increase in NE release in the IC and BLA following novel taste exposure (Guzmán-Ramos et al., 2012; Osorio-Gomez et al., 2021). Given that microdialysis has low temporal resolution, the observed increase in NE may result from slow changes in LC's discharge rate during the nonlicking periods, which are difficult to quantify in the current study. Moreover, it is well established that LC activation influences the sensory processing of auditory, olfactory, visual, and somatosensory stimuli (McBurney-Lin et al., 2019; Waterhouse and Navarra, 2019). Thus, increased LC-NE activity during states of stress, arousal, or focused attention may modulate taste perception and ultimately influence how animals respond to and remember new tastes.

In conclusion, we found that licking-related LC-NE dynamics were not affected by novel or unexpected taste stimuli, despite LC's established sensitivity to salient information. These findings suggest that LC neurons respond selectively to distinct forms of relevant information and may play a limited role in signaling features of taste. A more comprehensive characterization of LC-NE responses to taste stimuli will require the use of a broader array of tastants and a larger sample size. Expanding upon prior investigations of LC dynamics during consumption (Aston-Jones and Bloom, 1981; Sciolino et al., 2022), we also uncovered a connection between LC activity and lick bursts that was previously unrecognized. The exploration of the physiological function and circuit mechanisms underlying LC-NE dynamics during licking-related events presents a promising avenue for future research.

## Synthesis

Reviewing Editor: James Howard, Brandeis University Decisions are customarily a result of the Reviewing Editor and the peer reviewers coming together and discussing their recommendations until a consensus is reached. When revisions are invited, a fact-based synthesis statement explaining their decision and outlining what is needed to prepare a revision will be listed below. The following reviewer(s) agreed to reveal their identity: Elena Vazey, Federico Bermudez-Rattoni.

This manuscript has now been assessed by two reviewers. We have consulted regarding the concerns and suggestions they raised to arrive at a consensus on what we see as outstanding issues that must be addressed before the manuscript can be considered for publication. Here is a list of those comments/suggestions:

Major:

1. We suggest that the authors include more direct language about the operational definition of salience in this experimental paradigm as it relates to novelty/familiarity. We feel that this is especially necessary in light of the palatability difference between saccharine and citric acid to avoid any confusion or misinterpretation about palatability as a potential confound. In the context of your behavioral paradigm, is salience primarily tested in the 2x2 ANOVAs within tastant with Epoch and Familiarity as factors? In the context of this design, what would a typical signature of salience look like (e.g., interaction of Epoch and Familiarity on fluorescence signal?)? It may be helpful to move the results of these critical statistical tests that back up your primary claims from the figure legend(s) to the main text, so there is a more apparent connection between your experiment design and your claims regarding salience.

2. On page 13 it is stated: "We found that LC-NE dynamics during both citric acid exposures were like those observed during saccharine consumption (Fig. 1E)." We suggest that you formal test this comparison statistically to back up this claim, e.g. with a 2x2x2 ANOVA with tastant identity, epoch, and familiarity as factors.

3. Related to the previous point, there is a significant familiarity main effect for CA mentioned in the legend for Fig. 1E, but this effect is not really addressed in the main text. Does this main effect change the authors' interpretation of their claims of no salience signals for CA, and/or the claims of similar salience signal profiles for saccharine and CA? This issue could be partly resolved by the suggestion to directly compare saccharine and CA responses above, but should also be discussed with respect to the general claims about CA salience alone.

4. The authors state that burst analysis was restricted to saccharin trials as there were few bursts in the citric acid group. From the data in figure 1, they show an increase in post-lick response based on familiarity in citric acid but not saccharin. Would the linear regressions for citric acid reveal something different than saccharin? The authors could potentially answer this questions using data from all licks instead of restricting analysis to bursts.

5. It is noted that the sample size included in this experiment is relatively small. We recommend that the authors clarify the potential limitations of a small sample size by highlighting that an increased sample size would provide more comprehensive information for comparing stimuli and conditions in this design.

6. We are concerned that the passive tastant exposure procedure presented in Figure 3 is not really passive. The tastant is not unexpected and requires active engagement (licking) for delivery. As noted in the discussion actual passive exposure would involve infusing into oral cavity. How does the programmed taste delivery paradigm provide passive taste exposure if the pattern is always 10 water licks, 5 tastant licks? It may be beneficial to reframe this paradigm as providing unknown tastants. This would further fit with existing literature showing phasic LC responses to external cues are associated with either unexpected or predictive conditioned stimuli. Furthermore, phasic LC responses occur prior to motor responses and do not occur for stimuli in which no further motor response is required, e.g. in no-go tasks.

7. The cited studies showing an impact of downstream NE after novel tastants are limited in their temporal specificity. The authors should acknowledge that it is entirely plausible that the rebound activity in LC after licking bouts contributes to this previously observed noradrenergic modulation.

8. While the exclusive examination of Dbh neurons contributes valuable insights, the authors should recognize the potential implications of this selective evaluation on the overall assessment of LC activity. Focusing solely on Dbh neurons may lead to underestimating the broader LC dynamics. We recommend including information or analysis regarding the percentage of positive Dbh neurons within the LC to improve the study. This additional metric could provide a more comprehensive perspective on the distribution and involvement of Dbh neurons in the LC, offering readers a clearer understanding of the neurobiological landscape under investigation.

9. It would be beneficial to the paper's discussion to explore whether the LC activity or the NE release in the IC or amygdala is the salience comparator for novelty. It has recently been shown that direct photostimulation of the VTA or its cortical terminals can trigger cortical dopamine release, which enables the consolidation of novel taste recognition memories. These results suggest that the comparator threshold for novelty familiarity would be in the VTA-IC circuitry, not solely in the VTA. The paper results suggest cortical/amygdala NA release from elsewhere, but LC could be involved in comparing taste novelty and familiarity.

10. Several areas are involved in comparing novelty and familiarity, such as the VTA for dopamine, the NBM for acetylcholine, and the LC for noradrenaline. We suggest including in the discussion section an explanation of why the LC may be particularly important for taste and novelty discrimination.

11. The possibility of physical equivalence between the stimuli used should be discussed. Is there any literature suggesting the similarities between the sound, visual, or taste presentations?

12. The authors used only one gustatory concentration for appetitive or aversive stimuli. Could the LC activity respond differently using different gustatory, auditory, and visual stimuli concentrations?

Minor:

1. In figure 1 legend the authors refer to "GCaMP8f" whereas in the figure and throughout the paper they refer to "GCaMP8m". This discrepancy should be resolved.

2. The images in 1A of histology look very oversaturated. It may be helpful to decrease saturation or include some measure to show %LC cells with expression along with the placement validity of the optic fiber as LC. It would be an improvement to see this for the gustatory and visual/auditory animals as there were comparisons between responses across these groups of animals.

3. The figure legend for Figure 2 is out of order and does not match the figures, and all of the panels in Figure 2 are not referred to in the main text Results section. Also, the slope plus/minus error is the same for post-licking novel and familiar, is this correct?

4. In figure 3B there appears to be a rather sharp increase in response for saccharine presentation, but the authors report no significant effect. Is this potentially an artifact of including some below-zero baseline signal in the calculation of the saccharine response?

5. The authors show increased activity in LC-NE neurons after a licking bout that seems to last ~1s. What are the animals doing during that time? This may help explain/give credibility to the authors assumption that this LC-NE signal is reorienting the animal to its environment after attending to consumption.

6. On Page 3 it is stated "... as well as by neutral stimuli that predict them ..." We suggest the authors not refer to conditioned stimuli predicting appetitive or aversive outcomes as neutral.
