# Supplementary figures and images for: Locus Ceruleus Dynamics Are Suppressed during Licking and Enhanced Postlicking Independent of Taste Novelty
Source: eNeuro. 2024 Apr 16;11(4):ENEURO.0535-23.2024. doi: 10.1523/ENEURO.0535-23.2024 (PMC11036117; doi:10.1523/ENEURO.0535-23.2024)

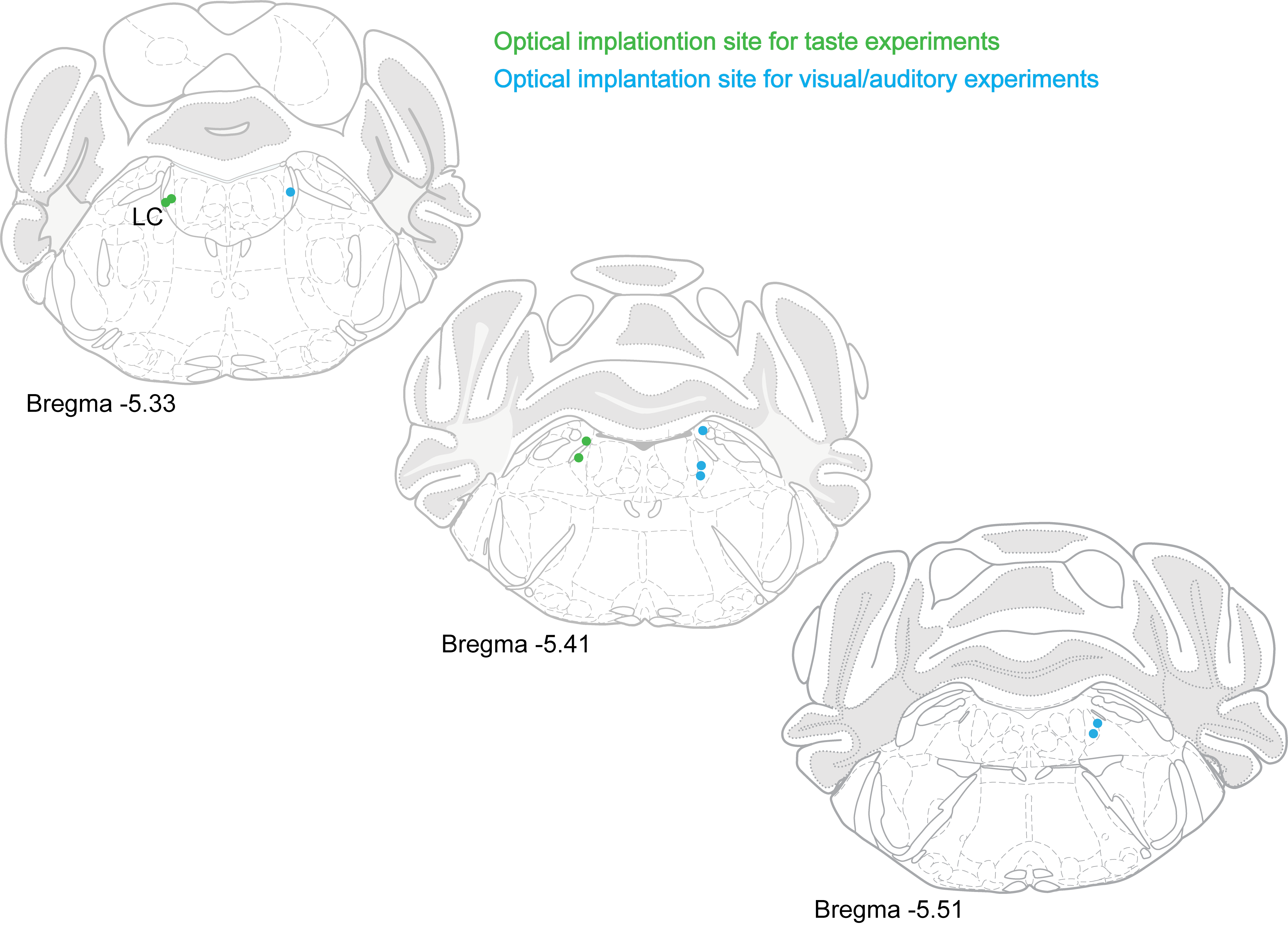

Supplement: Extended data Figure 1-1 — Fiber photometry recording sites relative to the LC. The location of the implanted optical fiber in the hindbrain of LCGCaMP/TdT mice. The ventral tip of the optical fiber is indicated by a dot for each subject. Mice from the taste experiment are shown as green dots (n = 4). Mice from the visual/auditory experiment are shown as blue dots (n = 6), which does not include two subjects due to technical issues with tissue collection that prevented the precise localization of the optical probe. The schematic shows coronal sections adapted from a mouse reference atlas (Paxinos and Franklin, 2019) using coordinates relative to bregma (mm). Mice from the different experiments are shown on the opposite hemisphere of the schematic for illustrative purposes. Download Extended data Figure 1-1, TIF file. [file eneuro-11-ENEURO.0535-23.2024-s001.tif]
